# Cardiovascular disease and type 2 diabetes in evolutionary perspective: A critical role for helminths?

**DOI:** 10.1093/emph/eow028

**Published:** 2016-09-25

**Authors:** Michael D. Gurven, Benjamin C. Trumble, Jonathan Stieglitz, Aaron D. Blackwell, David E. Michalik, Caleb E. Finch, Hillard S. Kaplan

**Affiliations:** 1 Department of Anthropology, University of California-Santa Barbara, Santa Barbara, CA 93106;; 2 School of Human Evolution and Social Change & Center for Evolution and Medicine, Arizona State University, Tempe, AZ 85287;; 3 Institute for Advanced Study in Toulouse, 21 allée de Brienne, 31015 Toulouse Cedex 6, France;; 4 Department of Pediatrics, University of California, Irvine School of Medicine;; 5 Department of Infectious Diseases, University of California, Irvine School of Medicine;; 6 Andrus Gerontology Center and Department of Neurobiology USC College, University of Southern California, Los Angeles, CA 90089 and; 7 Department of Anthropology, University of New Mexico, Albuquerque, NM 87131

**Keywords:** atherosclerosis, diabetes, helminths, ecological immunology, hygiene hypothesis, old friends hypothesis

## Abstract

Heart disease and type 2 diabetes are commonly believed to be rare among contemporary subsistence-level human populations, and by extension prehistoric populations. Although some caveats remain, evidence shows these diseases to be unusual among well-studied hunter-gatherers and other subsistence populations with minimal access to healthcare. Here we expand on a relatively new proposal for why these and other populations may not show major signs of these diseases. Chronic infections, especially helminths, may offer protection against heart disease and diabetes through direct and indirect pathways. As part of a strategy to insure their own survival and reproduction, helminths exert multiple cardio-protective effects on their host through their effects on immune function and blood lipid metabolism. Helminths consume blood lipids and glucose, alter lipid metabolism, and modulate immune function towards Th-2 polarization—which combined can lower blood cholesterol, reduce obesity, increase insulin sensitivity, decrease atheroma progression, and reduce likelihood of atherosclerotic plaque rupture. Traditional cardiometabolic risk factors, coupled with the mismatch between our evolved immune systems and modern, hygienic environments may interact in complex ways. In this review, we survey existing studies in the non-human animal and human literature, highlight unresolved questions and suggest future directions to explore the role of helminths in the etiology of cardio-metabolic disease.

## Introduction

Cardiovascular disease (CVD) accounted for 23% of all deaths in the USA in 2014, with at least half of these due to coronary artery disease (CAD), and another 5% to stroke [1]. Prevalence of type 2 diabetes mellitus (T2DM), which shares lifestyle and metabolic risk factors with CVD, is also rising in the USA and worldwide, and one-third of Americans are projected to have T2DM by 2050 [2, 3]. Although T2DM itself is a major health epidemic [3], it is often co-morbid with heart disease [4]. Although these chronic causes of morbidity and mortality are highly prevalent in industrialized populations, it is commonly believed by the public and specialists alike that these diseases were rare or absent throughout human evolutionary history. These afflictions of industrialized society are often viewed as classic examples of an evolutionary mismatch due to rapid environmental and lifestyle changes (‘modernization’) outpacing our evolved genetic heritage. According to this view, the widespread prevalence of CVD and T2DM today result from our being ‘Stone Agers in the fast lane’ [5]. In this review, we evaluate this notion, and then amend traditional views about the relevance of ancestral lifestyle factors on CVD and T2DM by exploring the role of our ‘old friends’—helminths [6].

Among past and contemporary hunter-gatherers, CVD and T2DM risk factors like obesity, hypertension, hypercholesterolemia and insulin resistance appear to be rare. Epidemiological surveys from the mid-20th century among! Kung hunter-gatherers [7], Central African pygmies [8], Australian aborigines [9], South African Bantu [10–12], Pacific Islanders [13, 14] and other rural, subsistence-level populations with minimal exposure to market economies [15, 16] support the notion that these risk factors are rare, and suggest that changes in diet, physical activity, other behaviors (e.g. smoking, alcohol consumption) and psychosocial stress alters the health of such populations in ways predicted by the mismatch hypothesis. Indeed, an experiment in the 1980s where ‘Westernized’ diabetic Australian aborigines were reintroduced to a traditional hunter-gatherer diet and lifestyle for seven weeks showed improvements in metabolic condition, including lower fasting glucose, triglycerides, blood pressure and weight loss [17, 18].

It is commonly believed that prehistoric humans might not have suffered from CAD, T2DM and other chronic diseases of aging because lifespans were short throughout most of human history, and because CAD and T2DM are not implicated as major causes of old age mortality. While old age mortality is certainly lower today (and modal ages of death about a decade longer) [19], there is no evidence that diseases like CAD and T2DM only exist today because of longer lifespans. Despite low life expectancies, hunter-gatherers and farming populations with limited access to medical care are likely to reach middle age and older adulthood if they survive early childhood. High infant and child mortality brings calculations of life expectancy at birth (*e*_0_) to 21–37 years for hunter-gatherer populations; however, given survival to age 15, the modal age of death for hunter-gatherers, farmers and even 18th century Europeans ranges from 68 to 78 years [20]. Thus, if CAD and T2DM are chronic diseases of aging universally expressed in all populations, then their manifestations should be readily observable among older members of subsistence-level societies.

Despite suggestive findings that CAD, T2DM and their risk factors are largely absent among contemporary pre-industrial populations, several considerations caution against concluding that these diseases represent a purely modern disease process, and are therefore absent in remote populations practicing traditional lifeways. First, computed tomographic (CT) scans of mummies spanning 4000 years from Egypt, Peru, the American Southwest and Aleutian Islands show evidence of probable or definite calcific atherosclerosis, and not just among well-fed elites [21]. The Tyrolean iceman (5300 y BP) of central Europe also exhibited calcification of both carotid arteries, the aorta and iliac artery [22]. Such calcifications are predictive of fatal vascular events in modern populations [23]. Second, the apparent absence of CAD and T2DM in extant populations could be due to methodological and logistic limitations. Given the demographic structure of most extant pre-industrial populations, sample sizes of older adults are small and subject to mortality bias, limiting statistical power and inference. Third, if case fatality rates are high among those afflicted with CAD and T2DM in the absence of healthcare, then cross-sectional surveys are unlikely to capture the short-lived cases, such as survival post-myocardial infarction (MI). Even the axiomatic claim that Greenland Inuit were free of atherosclerosis [e.g. 24] has been contested due to problematic mortality statistics [25]. Fourth, most studies of CAD and T2DM in remote populations focus on easily measured symptoms like blood pressure, blood lipid levels and anthropometrics rather than diagnosis from direct measures of atherosclerosis, which require greater technological sophistication, such as electrocardiogram (ECG) and CT, or detailed information on causes of mortality.

Yet even with these considerations in mind, converging evidence (see ‘case studies of cardio-metabolic disease in contemporary preindustrial human societies’ section) supports the early epidemiological surveys cited above and suggests that hunter-gatherers, horticulturalists and agro-pastoralists living under relatively traditional conditions do not experience CAD or T2DM. While more technologically sophisticated analyses and longitudinal data may suggest otherwise in the future, it seems unlikely that clinically relevant CAD or T2DM were ever frequent causes of morbidity and mortality among older adults in pre-industrialized societies. Lifestyle factors such as lean, high fiber diets free of processed foods, higher physical activity, minimal smoking and other behaviors are protective factors common to many preindustrial societies [5, 26]. Another important attribute common to these populations is the relatively high prevalence of diverse, frequently co-occurring infections [27]. To date, the cardio-protective role of infection against CAD or T2DM has not been seriously considered with rigorous studies. In fact, many infections can increase localized and systemic inflammation and are thereby believed to increase risk of CAD and T2DM, given epidemiological evidence linking inflammation to these conditions in industrialized populations [28–31]. Consistent with this notion, the reduction in infectious disease during epidemiological transitions of the past century not only reduced period mortality of older adults, but also reduced onset and fatality of chronic diseases including CVD among cohort survivors [32, 33].

**Figure 1.**
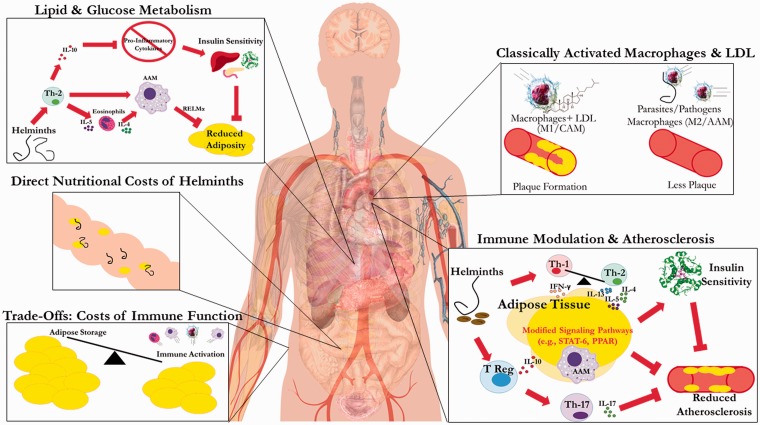
Summary of mechanisms by which helminths affect CAD and T2DM*.* ‘Lipid and Glucose Metabolism’: Helminths promoting Th2 immune bias induce systemic elevations in eosinophils and alternatively activated macrophages (AAMs or M2), especially in white adipose tissue. AAMs producing resistin-like molecule alpha (REMα) inhibit adipogenesis, while increased anti-inflammatory cytokines (e.g. IL-10) downregulate pro-inflammatory cytokines, increasing insulin sensitivity [34]. Together these factors reduce obesity and insulin resistance, lowering risk of T2DM. *Direct* ‘Nutritional Costs of Helminths’: Helminths can directly consume blood lipids, but may also decrease lipid levels by inhibiting their intestinal absorption, depending on species and density of infection. ‘Trade Offs: Costs of Immune Function’*:* Immune activation is energetically expensive, and results in increased RMR, which can lead to less adipose storage, or possible consumption of existing adipose tissue to generate eosinophils, macrophages and other immune components. ‘Classically Activated Macrophages (CAMs) and LDL’*:* In hygienic populations, CAMs or M1 cluster at the site of arterial injuries and bind with LDL cholesterol, resulting in calcified lesions that progress with repeated exposure. In presence of helminths, LDL is lower, and immunity is Th2-polarized with anti-inflammatory M2 macrophages recruited to fight infection; the net effect is decreased atherosclerotic lesion progression. ‘Immune Modulation and Regulation and Atherosclerosis’: Th2-biased immunity increases AAMs and regulatory T-cells, which release cytokines (IL-4, IL-5, IL-13, IL-10) that impact signaling pathways within adipose tissue (e.g. PPAR, STAT-6). T regulatory cells inhibit Th-17 responses (e.g. IL-17), and produce other anti-inflammatory cytokines (e.g. IL-10) that result in immunomodulation disfavoring atherosclerotic lesions, plaque vulnerability or insulin resistance [35]. Note: regular arrowhead suggests promotion, whereas flat arrowhead signifies inhibition

On the other hand, certain infections may have cardio-protective effects, either directly or indirectly through their effects on immune function and blood lipid metabolism (Fig. 1). Intestinal helminths are lipophilic and absorb lipids either from their host’s gut contents or blood stream, either of which could reduce their host’s circulating lipids and thereby minimize accumulation of plaques in vasculature [36]. Additionally, endo-parasites like helminths represent a significant energetic cost from immune activation that increase resting metabolic rates, further limiting adipose tissue deposition [37, 38]. These helminths were likely a prevalent source of infection over human evolutionary history. Their relative absence in modern, urban populations has likely contributed not only to the growing prevalence of auto-immune conditions associated with the ‘hygiene’ and ‘old friends’ hypotheses [6, 39], but may also contribute to aberrant inflammation and immune dysregulation that underlies many chronic non-communicable diseases like CAD and T2DM.

Historically, in modern urban populations, the decline in infections with improved public sanitation, access to clean water and hygiene, and vaccines often occurred concurrently with changes in diet, physical activity and other lifestyle behaviors, making it difficult to tease apart the separate contributions of each factor. Whether it is the absence of helminths alone, or more likely, in combination with standard metabolic risk factors like smoking, sedentary lifestyle and hyper-caloric diet that has the largest effect on CAD and T2DM progression remains to be determined. If minimal CAD and T2DM in traditional populations were only due to high physical activity, low cholesterol, protective diet, minimal obesity and the absence of other well-known risk factors, then further study of these populations as they undergo socioeconomic transformation might not yield new scientific discoveries that could ultimately benefit biomedicine. However, if helminths and other infections have cardio-protective roles, then new insights will be gained by focusing on populations currently under-represented in biomedical research.

In this review, we first discuss the role of inflammation and immune activation in CAD and T2DM etiology and progression (‘The critical role of inflammation in cardio-metabolic disease’ section), since one key pathway by which infections can alter risk is through immune system modulation. We next describe the role of helminths and other infections in human populations (‘Infections and human evolution’ and Harmful effects of infection and CAD T2DM’ sections ), and several ways in which their presence may mitigate CAD and T2DM (‘Cardio-metabolic protective effects of helminth infection’section 5). We evaluate existing experimental evidence from animal models and observational evidence from four human populations living a traditional lifestyle who have been studied intensively (Orumo of Ghana, Flores of Indonesia, Tsimane of Bolivia and Kitava of Trobriand Islands) (‘case studies of cardio-metabolic disease in contemporary preindustrial human societies’ section). Throughout the paper we focus more on CAD than T2DM given two recent reviews examining the role of helminths on T2DM [34, 35]. We conclude by proposing future research directions for evolutionary medicine (‘Future prospects’ section).

## The Critical Role of Inflammation in Cardio-Metabolic Disease

In addition to genetic risk factors and modifiable lifestyle behaviors, the immune system has been increasingly implicated in CVD and T2DM [40]. In particular, recent attention has helped redefine atherosclerosis as an inflammatory disease. Inflammation is the generic innate immune response to pathogens, damaged cells, and other irritations, whose function is to combat initial assault and to initiate tissue repair [40]. Acute inflammation occurs in response to bacterial and viral infection, and injury. Repeat activation and/or chronic inflammation over months or years can be destructive to tissues and lead to fibrosis, a condition more common in the presence of persistent disorders like asthma, tuberculosis, chronic periodontitis and hepatitis, rheumatoid arthritis and inflammatory bowel disease. In urban environments, chronic low-grade inflammation is prevalent among obese adults with excess lipids in adipose tissue and the liver [41], and among chronic smokers [42, 43]. Different sources of inflammation may vary in their downstream effects on vascular plaque formation; ‘infection-induced’ inflammation, a prominent source of inflammation in pre-industrial societies, may be less relevant than chronic low-grade inflammation from smoking, obesity and other processes (e.g. psychosocial stress) in the development of atherosclerosis [44, 45].

Inflammatory processes are implicated in all stages of CAD and T2DM [46–48]. With respect to CAD, the critical role of inflammation in mediating the pathogenesis of atherosclerotic plaques and MI has been well established for several decades, with both innate and adaptive immunity responding to self-antigens in the atheromatous plaque [49]. Most of these self-antigens are considered to be derived from trapped Apolipoprotein-B, oxidized LDL and lipoprotein remnants in the sub-endothelial intima, which accumulate in high velocity areas of arterial branching and when plasma LDLs are high [49, 50]. Indeed, immune cells such as macrophages, T cells, dendritic cells, and mast cells are all known to infiltrate atherosclerotic plaques [51]. Plaque antigens stimulate recruitment of monocytes from the blood to pass through the arterial endothelium and form macrophages to engulf the lipids that then transform into lipid-laden foam cells. The critical feature of plaque formation is that the lesion does not resolve, but instead a pro-inflammatory environment is created, which in turn recruits more monocytes, and a lipid-rich necrotic core forms from dead macrophages and other immune cells [52, 53]. Atherosclerosis is produced, both additively and in interaction, by a lipid-rich systemic environment and increased chronic, pro-inflammatory immune activation.

Inflammation is also recognized in the etiology of insulin resistance, metabolic syndrome and T2DM [54–58]. Consistent with the causal etiology described above, prospective studies show that high levels of inflammatory biomarkers such as high sensitivity C-reactive protein (hs-CRP), interleukin-6 (IL-6) and tumor necrosis factor alpha (TNF-α) are related to CAD onset, progression and CAD-related mortality [59–63]. Similarly, hs-CRP and other inflammatory biomarkers predict onset and progression of obesity, metabolic syndrome and T2DM [46]. In population-based studies, such as the Multi-Ethnic Study of Atherosclerosis, Atherosclerosis Risk in Communities and West of Scotland Coronary Prevention Study (WOSCOPS), inflammatory markers (e.g. IL-6, CRP), are strongly associated with diabetes risk [64–67]. A recent meta-analysis confirms the consistent dose-response relationships between IL-6, CRP and diabetes risk [66, 68]. The effects of low-grade inflammation, as measured through serum levels of various biomarkers in epidemiological studies, are largely independent of more traditional risk factors like hypertension, obesity and hypercholesterolemia, suggesting alternative moderators (e.g. infection) influencing the link between inflammation, CAD and T2DM etiology. Similarly, autoimmune conditions with excess inflammation such as rheumatoid arthritis, lupus, and granulomatosis are also risk factors for atherosclerosis [69].

## Infection and Human Evolution

Throughout history, human populations were exposed to an array of pathogens, many of which were common to other wild primate species [70]. Ancestral humans may also have been exposed to additional pathogens and parasites due to the consumption of meat and fish [71]. Phylogenetic evidence for several pathogens, including smallpox, *Plasmodium falciparum*, and *Mycobacteria tuberculosis* suggests a pre-agricultural history of exposure [see review in 72]. Sexually transmitted diseases also likely have a long evolutionary history among humans [73]. Antibodies to viral infections, such as herpes simplex, Epstein-Barr and varicella-zoster virus (VZV) have been documented in isolated Amazonian groups, along with cytomegalovirus (CMV), intestinal helminths, herpes simplex viruses, hepatitis B and arboviruses [74, 75].

Strong evidence suggests that helminths have coexisted with humans for millennia and represent a major feature of early human disease ecology [see 76 for review]. Non-human primates are widely infected with helminths, and infection with multiple species of soil-transmitted intestinal parasites has been documented in remote Amerindian populations [76–78]. Macro-parasites such as *Enterobius vermicularis* (pinworm) and hookworms (*Necator americanus* and *Ancylostoma duodenale*) have been discovered in coprolites from 7 to 10 kya [79, 80]. Throughout human history, helminths burdens have likely fluctuated. For example, sedentism and proximity to domesticated animals from the introduction of agriculture may bring about a higher parasitic burden [81, 82]. However, it is likely that until relatively recently very few humans had no helminth exposure whatsoever. Helminths have complex life cycles within human hosts, passing through numerous host tissues, and with intricate survival strategies that involve not only thwarting host immunity, but also competing with other helminths for host resources and creating a favorable niche by host manipulation [83, 84]. This long history suggests that human immune systems have co-evolved with helminths and may occasionally produce maladaptive outcomes under the novel conditions introduced in recent human history.

During the last 150 years, changes to the water supply, sanitation and public health infrastructure have lowered exposure to infectious diseases [85, 86], and immunizations and medical interventions have further reduced the prevalence of infectious diseases and their harmful impacts. Those changes in pathogen exposure and treatment have been accompanied by increased processed food consumption, tobacco and other drug use, reduced physical activity, higher rates of depression [87] and lower fertility, all of which may have complex effects on health outcomes.

## Harmful Effects of Infection on CAD and T2DM

Despite the unresolved causal role of systemic inflammation on CAD and T2DM risk [88, 89], there is increasing evidence that infection can generate localized inflammation in coronary arteries [28, 29, 90, 91]. There is also increasing evidence for the roles that viral and bacterial pathogens play in the etiology of atherosclerosis via inflammation [30, 92, 93]. An infectious organism can contribute to inflammation within a blood vessel, either directly by infecting vascular cells and activating innate immune responses, or indirectly by inducing inflammation at a nonvascular site such as the lungs in the case of *Chlamydia pneumoniae*.

In support of the direct route, viruses such as CMV, and bacteria such as *Helicobacter pylori* have been found in human and mouse atherosclerotic plaques [30]. Epidemiological studies also show an association between infection and atherosclerosis. For example, CAD is associated with the number of positive serologies against common infections (e.g. CMV, hepatitis A virus, HSV-1 and 2) after adjustment for traditional CAD risk factors [94]. In a prospective follow-up, individuals with five positive exposures were six times as likely to have MI or die than those with one exposure [95]. Other results, however, are inconsistent with a causal link between pathogen burden and atherosclerosis; clinical trials focusing on patients with CAD are unable to demonstrate long-term benefit to antibiotic interventions against *C. pneumoniae* [96–98]. Another possibility is that infections and immune activation increase oxidative stress [99, 100], which damages arterial epithelium, oxidizes lipids and leads to plaque formation [101].

The role of infection in the etiology of T2DM also has a growing body of support [102]. Hepatitis C virus appears to be directly involved in the development of insulin resistance [31, 103, 104]. Individuals infected with *H. pylori* are also at greater risk of developing T2DM [105, 106], although causality has not yet been established. Although *H. pylori* is associated with a 2.7 fold increase in risk of developing T2DM, other infections such as HSV1, VZV, CMV and *T. gondii* are not associated with increased risk [106].

## Cardio-Metabolic Protective Effects of Helminth Infection

Despite the systemic pro-inflammatory environment fostered by some bacterial and viral infections, other infections might offer protection against CVD and T2DM. A number of animal models provide evidence of protective effects of one type of parasitic infection—helminths (see Table 1). These include mostly intestinal geohelminths such as hookworm and roundworm, but may also include water-borne helminths such as schistosomes and insect-borne filarial helminths such as *Wuscheria bancrofti*. The notion that helminths in particular might offer protection against atherosclerosis was first proposed in 2005 by the Israeli physician, Eli Magen [124]. A small but growing number of studies are leading to productive directions for testing this hypothesis. For example, in apoE2/2 mice infected with *Schistosoma mansoni*, atherosclerotic lesions in the aortic arch and brachiocephalic artery were reduced by half compared to uninfected controls with the same diet [107, but see 108]. Another study reported a 60% reduction in aortic lesions in mice inoculated with a glycoprotein (ES-62) secreted by the filarial nematode *Acanthocheilonema viteae* [109]. Experimental infection of mice with the nematode *Nippostrongylus brasiliensis* lowered obesity and blood lipid levels, increased anti-inflammatory and immune regulatory activity, and improved insulin sensitivity [110]. Other murine studies show similar effects of helminths on a number of metabolic risk factors relevant to both CAD and T2DM [35, 108, 111, 112].

**Table 1.** Summary of all studies relating helminth infection to cardiometabolic indicators of relevance to CAD and T2DM

Helminth type	Species	Transmission	Primary infection site	Host species	Population	Major finding	Citation
Filarial Nematode	*A. viteae (glycoprotein only)*	Tick	Lymphatic system	Mouse	Lupus model	Atherosclerotic lesions reduced by 60%	[109]
Filarial Nematode	*Wuchereria bancrofti*	Mosquito	Lymphatic system	Humans	CURES study	Negative association between lymphatic filariasis and diabetes	[111]
Nematode	*N. americanus/ Ascaris lumbricoides*	Soil	Small intestine	Humans	Tsimane, Bolivia	Helminth infection associated with higher V02max	[122]
Nematode	*N. americanus/ A. lumbricoides*	Soil	Small intestine	Humans	Tsimane, Bolivia	Helminths unrelated but IgE and CRP/IL6 associated with lower blood lipids	[119]
Nematode	*T. trichiura/ N. americanus/ A. lumbricoides*	Soil	Large/small intestine	Humans	Flores island, Indonesia	Lower BMI and less insulin resistance with more helminth infection	[116]
Nematode	*T. trichiura/ N. americanus/ A. lumbricoides*	Soil	Large/small intestine	Humans	Flores island, Indonesia	Lower BMI, WHR, total cholesterol, LDL cholesterol. No association between helminth infection and carotid intima media thickness.	[120]
Nematode	*N. brasiliensis*	Soil	Small intestine	Mouse	IL-4 reporter gene	Lower obesity and blood lipids, improved insulin sensitivity	[110]
Nematode	*N. brasiliensis*	Soil	Small intestine	Mouse	RIP2-Opa1KO, STAT6 or IL13 deficient	Reduced weight and improved glucose metabolism	[112]
Nematode	*Ancylostoma ceylanicum*	Soil	Small intestine	Golden Hamster	Golden Hamster	Elevated VLDL, LDL, lower HDL	[123]
Nematode	*T. trichiura/ N. americanus/ S. stercoralis*	Soil	Large/small intestine	Humans	Shipibo, Peru	Negative association between egg count and HDL	[118]
Trematode	*S. mansoni*	Water	Mesenteric veins	Mouse	ApoE-knockout (-/-)	Atherosclerotic lesions reduced by half	[107]
Trematode	*S. mansoni (eggs)*	Water	Mesenteric veins	Mouse	ApoE deficient	Lower cholesterol	[111]
Trematode	*S. mansoni (soluble egg antigen)*	Water	Mesenteric veins	Mouse	C57BL/6 wild-type, LDL-/-	Reduced plaque size, progression, and intraplaque inflammation	[121]
Trematode	*S. mansoni*	Water	Mesenteric veins	Humans	schistosomal hepatic fibrosis patients	Low blood lipids, low atheroschelrosis	[113]
Trematode	*S. japonicum*	Water	Mesenteric veins	Humans	Rural China	Lower blood glucose, HbA1c, less insulin resistance, triglycerides and LDL	[115]
Trematode	*O. felineus*	Fish	Biliary tract	Humans	Russia, Khanty- Mansiisk region	Lower cholesterol, less fatty streaks, fibrotic plaques, and lesions on aortic surface, lower atherosclerosis	[117]
Trematode	*S. mansoni (eggs)*	Water	Mesenteric veins	Mouse	ApoE deficient	Lower cholesterol, no reduction in atherosclerosis	[108]

Several studies show similar associations in humans (Table 1). Reports include minimal clinical atherosclerosis in patients with schistosomal hepatic fibrosis [113], lower levels of T2DM with lymphatic filariasis [114], and lower blood glucose, glycated hemoglobin (HbA1c), insulin resistance, triglycerides and LDL with prior *Schistosomiasis japonicum* infection [115]. The Indonesian ImmunoSPIN project, detailed in ‘Ende of Flores Island’ section, has found that helminth infections are associated with greater insulin sensitivity [116]. An autopsy study of 319 cadavers in the Khanty-Mansiisk region of Russia measured both *Opisthochis felineus* worm burden and area of atherosclerotic lesions in the thoracic and aortic arteries [117]. Fatty streaks, fibrotic plaques and complicated lesions were inversely related to the number of worms per infected liver and were most common in uninfected individuals.

Below we outline several possible routes, summarized in Figure 1, by which helminths could be associated with lower CAD and T2DM risk.

### Blood lipids and energy restriction

Intestinal helminths are known to reduce energy intake and to be associated with anemia, poor nutritional status and micro-nutrient malabsorption [125–127]. In addition to interfering with host nutrition through altering consumption and absorption, helminths can also affect blood glucose and lipid levels directly. Many pathogens rely on blood glucose for energy [128, 129] and pathogen-induced immune activation is costly, requiring significant increases in resting metabolic rate and glucose utilization [38, 130, 131]. Increasing evidence suggests that host lipids are manipulated by, and allocated to pathogens. Helminths may regulate host lipid metabolism by stimulating a decrease in total cholesterol levels [107], particularly low-density lipoprotein (LDL) and Apolipoprotein B [132]. Several mechanisms may account for these decreases. Lipids mediate and are used by innate immune responses [133]. Helminths and protozoans (e.g. giardia) cannot synthesize their own lipids, and so consume and metabolize host lipids to generate phospholipid membranes [132], exploiting the host lipidome for their own survival and reproduction [84, 134, 135]. Other pathogens, including bacteria such as *H. pylori* and *M. tuberculosis* also exploit host lipids for their own growth, maintenance and signaling [136]. Similar findings have been obtained for enteroviruses through different mechanisms [137] and for dengue [138]. Parasitic worms may also lower LDL levels by regulating innate antibodies to cholesterol [132]. About one third of LDL turnover is attributed to the effects of these naturally occurring antibodies to cholesterol [139–141].

Relatively few studies have related infection to blood lipid levels. A variety of blood lipids (total cholesterol, LDL, very low-density lipoproteins (VLDLs), high-density lipoproteins (HDLs), triglycerides), as well as albumin and glucose, are lower in sheep infected with the liver fluke *Fasciola hepatica* compared with uninfected sheep [142]. In humans, an inverse association between HDL-C and the density of infection by several parasitic worm species (hookworm, *Strongyloides stercoralis*, *Trichuris trichiura*) was first documented among Amazonian Shipibo of Peru [118]. Hospital patients in Chandigarh, India, showed lower HDL-C if infected with entamoebic and giardia parasites [132]. Among elderly US Latinos, individuals infected with CMV, HSV and VZV had lower LDL (though not significantly) than their uninfected counterparts [106]. Among Tsimane Amerindians [Box 2], total cholesterol was 10 mg/dl lower among individuals with elevated CRP and IL-6, and 19 mg/dL lower among those with elevated IgE after controlling for age, sex, body mass index (BMI) and hemoglobin [119]. New unpublished analyses further show that eosinophil counts are inversely associated with BMI, total cholesterol, LDL, HDL, blood glucose and triglycerides. Hookworm infection and higher eosinophil counts are also associated with lower systolic BP. Resting metabolic rate is also higher among adults with active helminth infection [38].

Because oxidized LDL cholesterol is implicated in inflammatory cascades leading to endothelial dysfunction, plaque maturation and rupture, some have argued that heart attacks and other events stemming from atherosclerosis would be rare if LDL could be maintained <70 mg/dl [143]. However, the extent to which this low LDL level could be achieved with an omnivorous diet in the absence of parasites or statins is debatable.

### Regulation and modulation of immune function

One hypothesis linking parasitic infection to CAD risk is that helminths may attenuate atherosclerosis through interactions with host defenses [124]. Helminths do not simply evade host immune defenses, but instead modulate and regulate immune response in self-favoring ways to create niches that optimize their own survival and reproduction. Helminths increase anti-inflammatory T helper cell (Th)-2 type responses, increasing eosinophils, IL-4, and other Th-2 cytokines. Th-2 polarization induces ‘Alternative activation’ (or M2) macrophages, whereas Th-1 polarization induces more ‘classical activation’ (or M1) macrophages [144]. M1 macrophages are generated by pro-inflammatory factors like IFN-γ, or Toll-like receptor activation and secrete cytokines and chemokines promoting inflammatory responses. Polarized Th-2 immune activation associated with helminth infection modifies cytokine profiles, whereby anti-inflammatory IL-4, IL-10 and IL-13 protect vessel walls from oxidized LDL-induced monocyte injury in the endothelium, and downregulate fibrinogen synthesis [145]. Th-2 activation may also modulate responses to heat shock proteins, *C. pneumonia*, and cytomegalovirus, and downregulate monocyte activation, each of which has been tentatively linked to atherosclerosis [124]. M2 macrophages are induced by IL-4 or IL-13 and provide signals for tissue repair, wound healing and fibrosis. Overall, helminths and their anti-inflammatory effects are expected to reduce inflammation at sites of vascular damage, inhibit LDL-induced monocyte-endothelial damage, and thereby inhibit atherosclerotic lesion formation, and potentially subsequent plaque erosion and rupture.

Helminths also induce high levels of the antibody immunoglobulin-E (IgE), which binds to Fc receptors on surfaces of mast cells and basophils, and stimulates Th-2 responses. Levels of total IgE are >160 times greater in non-Western human populations which commonly experience helminth infection than in the USA [146]. A recent case-control study showed that adults with selective IgE deficiency (<2 IU/ml) had higher rates of arterial hypertension, peripheral vascular disease, ischemic heart disease, and carotid stenosis than matched controls [147].

In addition to fostering anti-inflammatory activity, helminths regulate Th-1 and Th-2 responses by favoring greater regulatory T-cells which produce down-modulatory cytokines IL-10 and TGF-β, and other immune-modulatory mechanisms [148]. This prevents clearance of the parasites by an immune system that might otherwise operate at full potential [83]. Immune regulation may therefore reduce recursive inflammatory and autoimmune-like Th-1 responses associated with many stages of atherosclerosis and insulin resistance, and temper the collateral damage of pro-inflammatory responses by fostering concomitant anti-inflammatory activity.

Helminths have been linked to T2DM through similar immune pathways. Mice infected with helminths inducing eosinophilia, elevations in IL-4, IL-13 and other cytokines associated with alternative activation of macrophages (M2) in white adipose tissue (e.g. IL-4, IL-13) show improved glucose tolerance and reduced fat mass [110]. Mice fed high-fat diets in the absence of helminths instead show greater M1 macrophage activation (e.g. by IL-6, TNF-α), which directly increased obesity, resistin release, and impaired glucose tolerance leading to greater insulin resistance. Even with diet-induced obesity, the eosinophilia and M2 macrophage activation in infected mice helped maintain glucose homeostasis.

Though lipids were discussed separately (‘Blood lipids and energy restriction’ section), the above description linking helminths to T2DM suggests much complex cross-talk between immune and metabolic pathways [149]. Macrophages and other immune cells infiltrate white adipose tissue, promoting a pro-inflammatory state in the presence of obesity (the M1 phenotype). Adipokines, tissue inflammation and other mechanisms beyond the scope of this review also implicate adipose tissue as critical in shaping obesity-induced peripheral insulin resistance. Inflammation-mediated insulin resistance might be beneficial for fueling immunity against acute bacterial infection, but becomes pathological when ‘sterile’ chronic low-grade inflammation is induced by obesity and other non-infectious origins [150]. In addition, immune modulation leading to Th-2 polarization may directly affect lipids and their metabolism [151]. For example, the redirection of lipids to immune function may result in lower accumulation of self-antigens against LDL and byproducts in the lumen and less immune activity directed towards those antigens. Consistent with this notion, atherosclerotic lesions and cholesterol levels are greater in Th-1 polarized mice deficient in Th-2 cytokine IL-4 or STAT-6 transcription factor [152].

The recent characterization and recognition of brown and beige adipose tissue has opened a new area of study. Beige fat cell activation induces thermogenesis and increases metabolic rate, resulting in weight loss and glucose homeostasis in mice, and possibly in humans [150]. Eosinophils and Th-2 cytokines are important for the biogenesis of beige or brown fat, while the activation of these adipose tissues is triggered by cold temperatures, exercise, and possibly through other mechanisms including IL-33 [153, 154]. Although the complex interactions between immune function and adipocytes have not yet been fully unraveled, there is growing evidence that immune cells interact with adipose tissue to modify glucose usage, lipid storage and metabolism.

### Other mechanisms

Other possibilities exist beyond lipid consumption and immune modulation. For example, parasites may divert immune resources, particularly monocytes and lymphocytes, towards infected tissues and away from the arterial lumen, decreasing formation of fatty streaks, fibrous plaques and complicated lesions. Suggestive of this possibility, Tsimane have very low levels of monocytes in circulation [37]. Along with the induction of a strong Th-2 bias, helminths may divert resources away from Th-1 type responses that aid in the continued growth of atherosclerotic plaques. Calcium is also critical to signaling in immune and other cell responses [155, 156], and chronic immune activation from infection may lower serum calcium, potentially resulting in less calcification of arterial plaques (though such a connection remains controversial) [157].

## Living in a Poly-Parasitic World

An early version of the hygiene hypothesis proposed that insufficient bacterial exposure in childhood affecting Th-1 development can bias individuals toward Th-2 mediated pathologies such as asthma and allergies. It was later proposed that Th-2 stimulating ‘old friend’ parasites such as helminths could help counteract Th-2 mediated pathologies by leading to a better regulated immune network [39]. It is likely that the combined suite of pathogens to which the host is exposed determines whether the net effect of infections is to delay or accelerate CAD and T2DM. Populations with helminths often show co-infection with multiple helminths inhabiting different tissues, and sometimes lower levels of giardia and other pathogens [84]. Interactive effects with gut microbiota are also likely to affect how helminths modulate immunity. Gut microbiota have been shown to be associated with energetic metabolism, inflammation and obesity, and to metabolize pro-atherogenic trimethylamine-N-oxide from red meat [158] and eggs [159], and thus has also been invoked as relevant to CAD and T2DM etiology [160]. Type 2 immunity provided by helminth infection inhibits inflammatory *Bacteroides* colonization and promotes protective *Clostridiales* in mice [161], while helminth-induced alteration of bacterial microbiota reduces allergic asthma [162]. Subsistence populations show a richer diversity of gut microbiota than market-integrated populations [163, 164] and helminth infections have been shown in one study to associate with increased microbiota diversity [165]. Whether these findings generalize to a broad range of populations remains to be seen, but it is likely that the joint composition of both microbiota and macrobiota may be important for maintaining host intestinal and immune homeostasis [166]. The robust finding from recent research among subsistence populations experiencing a greater diversity of pathogens shows minimal evidence for CAD and T2DM, and no evidence that higher levels of inflammation due to infection results in greater CAD and T2DM burden (see ‘case studies of cardio-metabolic disease in contemporary preindustrial human societies’ section).

One possibility is that having a greater diversity of both Th-1 and Th-2 stimulating pathogens might lead to less pathological relationships between certain infections and CVD, such as the oral pathogen underlying periodontitis, *Porphymonas gingivalis* [167]. Additionally, diet, exercise and metabolic factors likely interact with the suite of infections, and resultant inflammation in affecting CVD and T2DM risks. Inflammation may only be pro-atherogenic and pro-diabetic in the context of excess energy, adiposity and high serum lipid levels, as is more commonly found in sedentary urban environments.

## Case Studies of Cardio-Metabolic Disease in Contemporary Preindustrial Human Societies

### Ende of Flores Island

**Figure 2.**
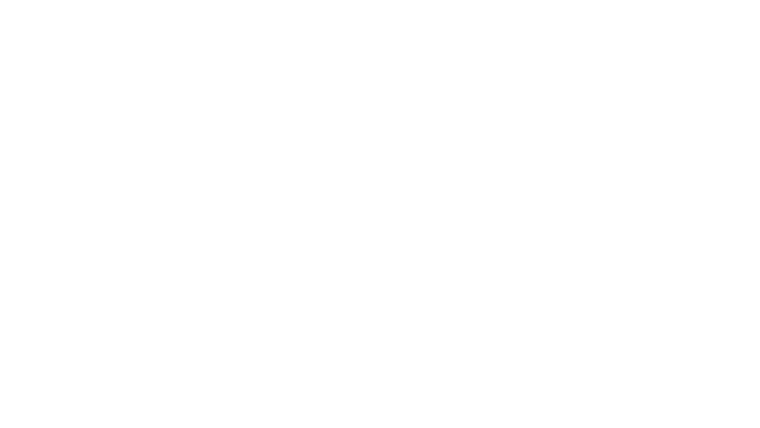
Contemporary preindustrial study populations. **(a)** Three Ende women at a health clinic (Photo credit: Aprilianto Eddy Wiria). **(b)** Two elderly Tsimane from a remote Tsimane village along the Maniqui River (Photo credit: Michael Gurven). **(c)** An older Bimoba woman in upper east Ghana (Photo credit: David van Bodegom). **(d)** Elderly Kitava man and woman (Photo credit: Staffan Lindeberg). Note: all photos were obtained with permission and consent for use

**Figure 3.**
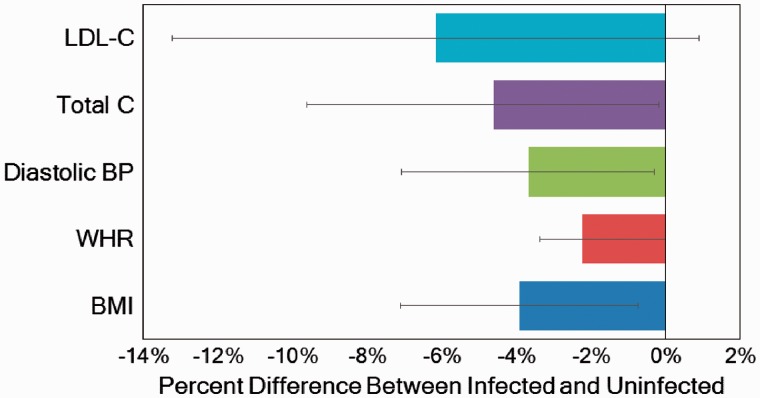
Difference in cardiometabolic risk factors between Ende infected (*n* = 446) and uninfected (*n* = 229) with at least one soil-transmitted helminth. Cross-sectional representative sample of adults age 18+ in a semi-urban area of Nangapanda on Flores Island were collected from May to August 2009. Most prevalent helminths include *N. Americanus*, *A. lumbricoides*, *T. trichiura*. Error bars are the 95% CI for the mean difference. Results are similar but attenuated when adjusting for age, sex and BMI. Based on [120]

In the rural and semi-urban Nangapanda area of Flores Island, Indonesia, Maria Yazdanbakhsh, Aprilianto Wiria and colleagues have studied the role of helminths on immune function and chronic disease among Ende farmers as part of the ImmunoSPIN project [116, 120, 168] (Fig. 2a). Adults are relatively lean, but blood lipid levels and blood pressure are high in comparison to other subsistence-level populations (e.g. mean LDL is ∼123 mg/d; mean systolic/diastolic pressure: 130/77 mmHg). Helminth infection (especially *N. americanus, A. lumbricoides, T. trichuris*) is common, and associated with higher IgE levels. They find that helminths are associated with lower T2DM and CAD risk factors. Those with helminth infection have lower BMI, smaller waist-to-hip ratio, and lower LDL and total cholesterol compared with uninfected individuals (Fig. 3). Moreover, having more helminth co-infections is associated with lower BMI, WHR and total cholesterol among those infected. Each additional helminth co-infection is associated with 4.9 pmol/l lower insulin, independently of their inverse relationship with BMI [116]. Higher IgE is also associated with lower HDL, total cholesterol and fasting blood glucose.

### Tsimane of Bolivia

Tsimane are forager-horticulturalists of the Bolivian Amazon (pop’*n* ∼ 16 000) studied by the Tsimane Health and Life History Project since 2002 (Fig. 2b). Tsimane experience higher pathogen burden than Western populations, including intestinal and vector-borne parasites, fungi, bacteria, viruses and protozoa. They also show higher levels of immune activation and inflammation, measured by white blood cell counts, erythrocyte sedimentation rate, CRP and IL-6 [169]. Eosinophilia (>500/ul) is abundant (87%), as is monocytopenia (<2%) (93%) [37]. Systemic immunity shows indications of chronic activation from parasitic infection, with serum immunoglobulins two orders of magnitude higher than among US adults, including IgE; Tsimane mean IgE is 10 719 (±251) IU/ml compared with US reference ranges (<100 IU/ml).

**Figure 4.**
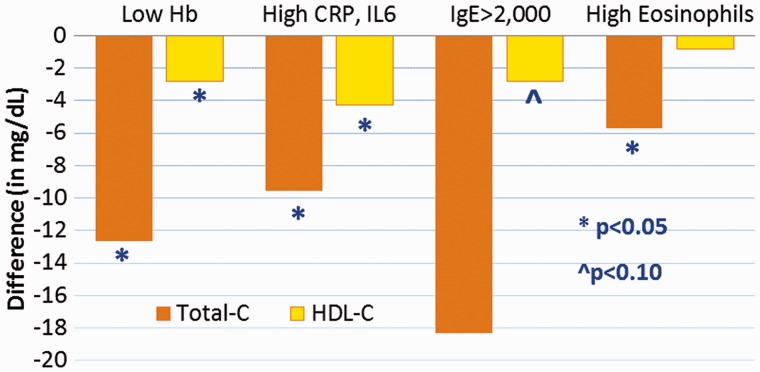
Relationships between indicators of infection and immune activation on blood lipids. Sample of 418 adults age 20+ from 17 Tsimane villages in 2004, collected as part of the Tsimane Health and Life History Project. Low hemoglobin, high CRP and IL-6, high IgE and eosinophil count are all associated with lower total blood cholesterol, and to some extent with lower HDL cholesterol. Results based on multiple regression analyses of total-C (*n* = 345) and HDL (*n* = 318) that also control for age, sex, BMI. Low Hb refers to first quartile, high eosinophils refers to fourth quartile. Based on [119]

Yet despite their pro-inflammatory state, there is no robust evidence of MIs. A sample of 860 echo-cardiograms of adults age 40–85 revealed only two cases of possible MI, as evidenced by wall motion abnormalities, and even those cases were considered dubious by the team of cardiologists that found CT-based evidence of atherosclerosis in human mummies (see ‘Introduction’ section). In a sample of 350 ‘verbal autopsies’ using the 2012 WHO instrument [170], only one case suggestive of MI was found, indicating that people in the U.S. are more than 50 times more likely to die from MI than Tsimane. In addition, hypertension is rare among Tsimane, and most adults over age 40 show no increase in blood pressure with age [171]. Tsimane also have very low prevalence of diabetes (<1.5%). Levels of total cholesterol (TC) and LDL are very low, with <2% of the population having levels above typical clinical cut-offs for TC (>240 mg/dl) or LDL (>130 mg/dl). This finding is noteworthy because Tsimane adults are not very lean, with 21% overweight (BMI between 25 and 30 kg/m^2^). Ongoing research relating infection to CAD and T2DM progression has shown protective effects on cholesterol, BMI and blood glucose (see text, Fig. 4).

### Bimoba of Ghana

The Bimoba, Kusasi, Mamprusi and Peul tribes of the upper east region in Ghana are subsistence farmers only recently undergoing an epidemiological transition (Fig. 2c). Ongoing studies since 2001 by van Bodegom, Westendorp, Koopman, and colleagues from Leiden University Medical Center have reported that these groups inhabit an infectious environment, rife with malaria, helminths, typhoid fever, and protozoans; these have led to selection for pro-inflammatory genotypes and strong innate immune responses [172, 173]. Among adults age 50+, CVD and T2DM risk factors are low: obesity is rare (<2%) and dyslipidemia is low (1–5%), but hypertension is somewhat prevalent (∼25%). T2DM is also rare (1% have glucose > 7 mmol/l). Direct evidence based on ECG and ultrasound measurement of ankle-brachial blood pressure suggests minimal overt CVD: myocardial infarcts and peripheral arterial disease are both rare (<1.2%, 2.8%, respectively), as are both myocardial ischemia-like changes and atrial fibrillation (11% and 0.3%, respectively) in comparison to age-matched USA and European comparison samples [174]. Although CVD and T2DM are rare, and infection highly prevalent, direct linkages between indicators of helminth infection and cardiometabolic health have not yet been studied.

### Kitava of Melanesia

The Kitava, subsistence horticulturalists of the Trobriand Islands, have been studied by Staffan Lindeberg, Johan Frostegård and colleagues since 1990 (Fig. 2d). The Kitava are lean and have low blood pressure and blood lipid levels [175]. Carbohydrates make up 69% of the diet, including yams, sweet potatoes, taro and fruit; fat, salt, cereal grain and dairy intake is low. ECGs and surveys revealed no indications of heart attack, stroke or angina pectoris, again suggesting that ischemic heart disease was minimal or absent in this population [176]. Plasminogen activator inhibitor-1 and other risk factors for thrombosis are also low [177]. Older adults also do not appear to show worsening age profiles of many CVD risk factors compared to younger adults (e.g. BMI, plasminogen activator inhibitor 1) [178]. Serum insulin and glucose levels are also low, especially among older adults, consistent with favorable insulin sensitivity [179]. Lindeberg and colleagues have argued that the physically active Kitava lifestyle free of processed foods is largely responsible for the lack of CVD and T2DM. Additionally, they suggest a possible relationship between infection and CVD based on serological evidence of treponemal bacterial spirochetes. Anti-treponemal IgM antibodies are highly prevalent among Kitava and attributed to subclinical yaws disease [180]. Treponemal infection can induce IgM autoantibodies to the epitope phosphorylcholine (PC), which were observed at higher levels among Kitava than a matched Swedish sample [181]. Because these anti-PC antibodies can inhibit uptake of oxidized LDL in macrophages, the presence of treponemal infection could be anti-atherosclerotic [180, 181]. Helminths are also prevalent among Kitava, but have not yet been investigated.

## Future Prospects

We have proposed that helminths may offer protection against CAD and T2DM due to their modulatory and regulatory effects on both immune function, and other risk factors such as blood cholesterol levels, metabolism and insulin resistance. Our focus on immune dysregulation as a central feature of CAD and T2DM is consistent with claims that atherosclerosis is an autoimmune disease [182]. Thus, helminth-induced Th-2 stimulation, anti-inflammatory activity, regulation and alternative macrophage activation can offer important protection.

Over a billion people worldwide are infected with at least one soil-transmitted helminth, though prevalence is confined largely to lower income countries lacking public health infrastructure [183]. Helminth eradication has therefore been a public health target, aimed at improving child growth, school performance and economic productivity, and host defenses against other infections (e.g. malaria, tuberculosis, HIV) [184]. The toll of helminth infection on the average lifespan has been estimated to be at least 4.7 disability-adjusted life years [183]. However, recognition that helminths may potentially reduce morbidity from CAD and T2DM, and other inflammatory diseases, should reduce this estimated burden, and at the same time, deworming campaigns could have harmful consequences later in adulthood [185].

To date, descriptions of the effects of ‘Westernization’ on increasing CVD and T2DM risk focus almost exclusively on changes in traditional Framingham risk factors [e.g. 27, 185]. More research is needed to better understand the varied and intricate proximate mechanisms briefly outlined above before developing interventions that could mimic the effects of helminths but without any harmful effects. There are many unanswered questions about how different helminths impact the host responses described here, and others, such as whether helminths influence all types of HDL equally. Although a new infection or exposure to helminth protein products may not reverse arterial calcification or other chronic processes, indications suggest that rapid changes in other CAD and T2DM risk factors are possible. For example, the Wu *et al.* [110] murine study described earlier showed that a single infectious episode of up to only eight days provoked sustained eosinophilia in adipose tissue, lowered blood glucose, increased insulin sensitivity and prevented excessive weight gain. Golden hamsters injected with *S. mansoni*-derived soluble egg antigen showed reduced blood cholesterol, and atherosclerotic plaque size and progression, partly by reducing the number of inflammatory monocytes, and reducing recruitment and accumulation of myeloid cells in the plaques [121]. An ongoing clinical trial in Indonesia (SUGARSPIN) is employing randomized double-blind, placebo-controlled experiments to better test causal relationships between helminths, insulin sensitivity and metabolic variables [187]. The mouse experiment introducing the anti-inflammatory glycoprotein ES-62 secreted by a filarial nematode that inhibited inflammation and protected against arthritis, asthma, and atherosclerosis highlights another avenue of potential intervention using helminth protein products [109]. Ongoing clinical trials are using self-limiting non-human parasites (e.g. *Trichuris suis*, a pig whipworm) or antigens from attenuated or inactivated human parasites to treat autoimmune disorders [188–190]. CAD and T2DM treatments based on similar principles could be tested in the future.

Other important questions remain. It will be important to explore how helminth exposure early in life versus adulthood might have differing consequences of various aspects of immune function and blood lipids, how duration of exposure affects outcomes, and whether antigens or helminth-derived products can substitute for live helminth infection. Also, helminths may have different effects on male versus female hosts. How helminths interact with bacterial and fungal microbiomes is also relatively unexplored but could have important consequences on host immunity [191].

Another area of future research is to expand the domain of genetic association studies. Amerindians show distinct human leukocyte antigen (HLA) expression at various MHC loci compared with other populations that show evidence of overdominant selection [192]. Although HLA-DR expression in macrophages and T-cells has been linked to plaque eruption and erosion [193], it is an open question whether allelic variation is of clinical significance. Genes affecting monocyte recruitment (e.g. CD14 receptor polymorphisms) [194], lipid transport (e.g. cholesteryl ester transport protein) [195], lipid oxidation, and modulation of the inflammatory response to oxidized lipids may also help explain differences in susceptibility of populations to developing atherosclerosis [196].

## Conclusion

Although CAD and T2DM are major contributors to mortality in urban populations, they may not have been significant causes of adult morbidity and mortality throughout most of human evolutionary history when infectious pathogens caused much of mortality and when inflammation was largely pathogen-driven. Evidence from past and contemporary subsistence-level populations suggests that CVD and T2DM risk factors like obesity, hypertension, hypercholesterolemia and insulin resistance are rare. Although there is evidence of atherosclerosis in HORUS studies of mummy CTs [21], these calcifications may not have had clinical symptoms severe enough to result in hard events like stroke or heart attack. Extant and historical subsistence populations may show evidence of arterial stiffening and calcification, but lower likelihood of plaque erosion, rupture and thrombosis. If subsistence-level populations were free of CAD and T2DM only because of minimal obesity, greater physical activity, low hypertension, low LDL and healthy diet, then we would have little to learn from their continued study. However, the relatively long co-evolutionary history of helminths and other pathogens with humans highlight potential mutualisms with beneficial effects on human health. Given the evidence summarized here, the recognition that most biomedical studies rely on pathogen-free laboratory models or pathogen-sparse Western populations suggest that there is still much to learn about CAD and T2DM etiology, progression, prevention and treatment.
